# Environmental Stewardship: A Conceptual Review and Analytical Framework

**DOI:** 10.1007/s00267-017-0993-2

**Published:** 2018-01-31

**Authors:** Nathan J. Bennett, Tara S. Whitty, Elena Finkbeiner, Jeremy Pittman, Hannah Bassett, Stefan Gelcich, Edward H. Allison

**Affiliations:** 1 Institute for Resources, Environment and Sustainability, University of British Columbia, Columbia, Canada; 2 School of Marine and Environmental Affairs, University of Washington, Washington, USA; 3 Center for Ocean Solutions, Stanford University, Stanford, USA; 4 Center for Marine Biodiversity & Conservation, Scripps Institution of Oceanography, University of California, San Diego, USA; 5 Hopkins Marine Station, Stanford University, Stanford, USA; 6 School of Environment, Resource and Sustainability, University of Waterloo, Waterloo, Canada; 7 Center of Applied Ecology and Sustainability, Pontificia Universidad Catolica de Chile, Santiago, Chile

**Keywords:** Environmental stewardship, Motivations, Capacity, Conservation, Environmental management, Sustainability

## Abstract

There has been increasing attention to and investment in local environmental stewardship in conservation and environmental management policies and programs globally. Yet environmental stewardship has not received adequate conceptual attention. Establishing a clear definition and comprehensive analytical framework could strengthen our ability to understand the factors that lead to the success or failure of environmental stewardship in different contexts and how to most effectively support and enable local efforts. Here we propose such a definition and framework. First, we define local environmental stewardship as the actions taken by individuals, groups or networks of actors, with various motivations and levels of capacity, to protect, care for or responsibly use the environment in pursuit of environmental and/or social outcomes in diverse social–ecological contexts. Next, drawing from a review of the environmental stewardship, management and governance literatures, we unpack the elements of this definition to develop an analytical framework that can facilitate research on local environmental stewardship. Finally, we discuss potential interventions and leverage points for promoting or supporting local stewardship and future applications of the framework to guide descriptive, evaluative, prescriptive or systematic analysis of environmental stewardship. Further application of this framework in diverse environmental and social contexts is recommended to refine the elements and develop insights that will guide and improve the outcomes of environmental stewardship initiatives and investments. Ultimately, our aim is to raise the profile of environmental stewardship as a valuable and holistic concept for guiding productive and sustained relationships with the environment.

## Introduction

The need to promote improved human-environment interactions through stewardship is ever pressing, which applies to terrestrial, marine, aquatic, and aerial environments in both rural and urban environments (Millenium Ecosystem Assessment 2005; Allsopp et al. 2009; Rockström et al. 2009; Chapin et al. 2010; Díaz et al. 2015; Davy et al. 2017). Many individuals, local communities, environmental groups, and governments around the world are taking and promoting actions to steward the environment. The term environmental stewardship has been used to refer to such diverse actions as creating protected areas, replanting trees, limiting harvests, reducing harmful activities or pollution, creating community gardens, restoring degraded areas, or purchasing more sustainable products. It is applied to describe strict environmental conservation actions, active restoration activities and/or the sustainable use and management of resources. Stewardship actions can also be taken at diverse scales, from local to global efforts, and in both rural and urban contexts. The global scale of many current environmental issues might lead to the perception that local actions can no longer meet these challenges. However, one way through which people get involved in promoting sustainability and in responding to external drivers of change, using their own expertise and knowledge, is through engaging in local environmental stewardship actions and initiatives. Thus, implicit in our framing of environmental stewardship throughout this article is a focus on the often-central role of local people in caring for the environment that they are proximal to, connected to and, in some contexts, that they depend on for subsistence needs and livelihoods.

Our focus on local stewardship also aligns with an increasing emphasis on local communities and resource users in conservation and environmental management policies, programs and practice globally, as evidenced in initiatives such as community-based conservation (CBC), community-based management (CBM), community-based natural resource management (CBNRM), indigenous and community conserved areas (ICCAs), integrated conservation-development projects (ICDPs), locally managed marine areas (LMMAs), “other effective area-based conservation measures” (OECMs), and urban stewardship initiatives (Barrett and Arcese 1995; Berkes 2004; Cinner and Aswani 2007; Govan et al. 2009; Krasny and Tidball 2012; ICCA 2013; Jupiter et al. 2014; Jonas et al. 2014; Riehl et al. 2015; Campos-Silva and Peres 2016). As these examples show, locally-oriented stewardship practices, policies and programs have emerged in fisheries, agriculture, forestry, protected areas, wildlife, ecosystem service, and water management across rural to urban environments. Fisheries management, for example, has seen a growing emphasis on the role, rights and responsibilities of small-scale fishers in stewarding local resources—as evidenced in programs such as Chile’s Territorial Use Rights Fisheries program (TURFs) (Gelcich et al. 2015), the rise of community supported fisheries programs globally (Brinson et al. 2011; McClenachan et al. 2014), the release of the global “Voluntary Guidelines for Securing Sustainable Small-Scale Fisheries” (FAO 2015) and increased funding of NGO programs that focus on small-scale fisheries (e.g., the Fish Forever Program (Barner et al. 2015)). In the agriculture sector, community supported agriculture initiatives—which reward farmers for stewardship-oriented practices—have emerged over the last few decades (Fish et al. 2003; Campbell et al. 2014; Raymond et al. 2016). Community-based forestry programs have grown in popularity since the 1980s, and have spread from the global south to the global North (McDermott and Schreckenberg 2009; Baynes et al. 2015). In urban environments, municipalities can support civic-led efforts or develop and promote initiatives such as community gardening, shellfish re-introductions, tree planting, invasive species removal, and conservation of soil, water and green spaces (Krasny and Tidball 2012; Connolly et al. 2014; Krasny et al. 2015). These are just a few examples to demonstrate that local environmental stewardship is promoted for diverse natural resources across all environments and geographies.

The academic literature provides many insights into environmental stewardship that might guide these local efforts. The phenomena of local environmental stewardship has been studied in numerous different contexts, including forests (English et al. 1997; Adhikari et al. 2007; Kilgore et al. 2008; Messier et al. 2015), freshwater (Shandas and Messer 2008; Kreutzwiser et al. 2011), grasslands and rangelands (Appiah-Opoku 2007; Squires 2012; Sayre et al. 2013; Henderson et al. 2014), rural agricultural landscapes (Worrell and Appleby 2000; Plummer et al. 2008; Ellis 2013; Gill 2014; Raymond et al. 2015), urban environments (Krasny and Tidball 2012; Connolly et al. 2014; Romolini et al. 2016), fisheries (Gray and Hatchard 2007; McConney et al. 2014; van Putten et al. 2014; Medeiros et al. 2014) and coastal or marine habitats (Sharpe and Conrad 2006; Friedlander et al. 2013; Silbernagel et al. 2015). These studies tend to focus their analysis either on a subset of the different factors that can support or undermine stewardship—for example, on ethics, motivations, capacity, institutions, networks, context—or simply on whether or not action is being taken to steward the environment. Few of these papers provide definitions of stewardship and those that do often focus either on the ethical dimension or simply on stewardship as behaviors or actions. To our knowledge there are no academic studies that provide a comprehensive definition and integrative analytical framework to bring together the different elements of environmental stewardship that have been discussed and examined across the literature. However, there are many existing frameworks for related concepts such as social–ecological systems, sustainable livelihoods, CBNRM, adaptive co-management, and environmental governance (Scoones 1998; Plummer and Fitzgibbon 2004; Tyler 2006; Ostrom 2009; Armitage et al. 2010), which can inform such an effort. In particular, these frameworks provide useful ways of thinking about the capacities and institutional factors that might support stewardship efforts.

In sustainability science, frameworks attempt to bring together the essential elements of a phenomena in order to facilitate descriptive, evaluative, diagnostic and prescriptive inquiries by diverse groups of interdisciplinary scholars on a topic of mutual interest (McGinnis and Ostrom 2012). The lack of an integrative framework for environmental stewardship limits our ability to systematically analyze case studies, build theory, and produce practical guidance on such questions as: How can local stewardship initiatives be designed or supported to be effective and appropriate in different contexts?; What enables or undermines the effectiveness of environmental stewardship?; or, How might external organizations, governments and consumers effectively promote or support local stewardship efforts? This paper thus fills a gap in the literature through presenting such a comprehensive definition and integrative analytical framework to structure future research and to help to improve efforts to support stewardship of the environment. To achieve this, we review and resituate insights from across the empirical and theoretical literatures on environmental stewardship, management and governance to understand and define the central factors that influence stewardship outcomes.

The paper is structured as follows. First, we provide a definition for local environmental stewardship. Next, we unpack the elements of this definition to develop an analytical framework. Finally, we discuss potential interventions and leverage points for promoting or supporting local stewardship and future applications of the framework to guide descriptive, evaluative, prescriptive or systematic analysis of environmental stewardship.

## Towards an Integrative Framework for Local Environmental Stewardship

Building on the broader body of work on this topic that is reviewed throughout this paper, we propose the following definition for local environmental stewardship:

Local environmental stewardship is the actions taken by individuals, groups or networks of actors, with various motivations and levels of capacity, to protect, care for or responsibly use the environment in pursuit of environmental and/or social outcomes in diverse social-ecological contexts.

**Fig. 1:**
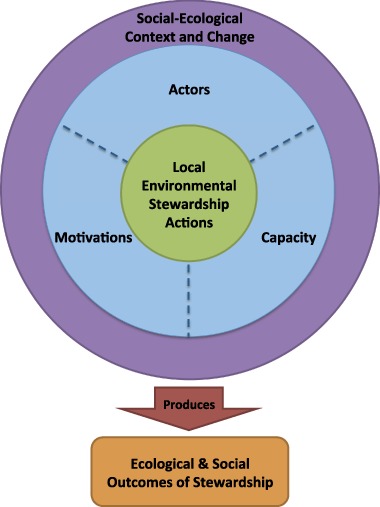
A conceptual framework for local environmental stewardship

In this definition, stewardship actions hinge on three central elements—actors, motivations and capacity—that are influenced by the social–ecological context and that converge to produce both environmental and social outcomes (Fig. 1). Below, we draw on a cross section of the literature on environmental stewardship, management, conservation, and governance from different contexts to unpack the elements of this definition and present an analytical framework for understanding local environmental stewardship.

### Actors: Individuals, Groups or Networks of Stewards

Stewardship actions are carried out by stewards—which can be individuals, groups, or networks of actors (Svendsen and Campbell 2008; Wolf et al. 2011; Bodin 2017). Individual stewardship actions, for example, might include daily decisions made by individual resource users regarding maintenance or restoration of soil, the management of vegetation, removal of invasive species, the quantity of marine resources extracted, the type of extraction practice used and its related environmental impact, or where harvest occurs depending on sensitivity or vulnerability of habitat. Stewardship actions can also be executed collectively by groups or communities to manage common-pool resources or common areas (e.g., urban community gardens) (Ostrom 1990, 1999; Cox et al. 2010; Krasny and Tidball 2012). This might even include collective decisions within cooperatives or communities to enforce more stringent conservation measures than mandated by the government (McCay et al. 2014). Which actors are involved in different stewardship actions largely depends on the scale and complexity of the issue as discussed below. In many cases, stewardship actions involve hybrid networks or multi-stakeholder partnerships that include public agencies, civil society organizations, funding bodies, NGOs, and local communities (Connolly et al. 2014; Finkbeiner and Basurto 2015; Romolini et al. 2016).

To understand how and why stewardship is or is not occurring, it is useful to understand the different individuals or configurations of actors across scales of organization who are initiating and driving local stewardship initiatives (Ostrom 2010; Guerrero et al. 2014; Alexander et al. 2015; Sayles and Baggio 2017). It can also be instructive to explore the actual, appropriate and desired allocation of rights, roles and responsibilities to different actors in the stewardship of local resources or areas. For example, in many places traditional harvesters or indigenous groups have legal or historical tenure or rights to local areas or resources—and, indeed, have often been the effective custodians of these resources (Berkes 1999; Gavin et al. 2015; McMillen et al. 2017). The assignation of rights to and support for stewardship to these local communities who are most dependent on local resources might be deemed most appropriate by some actors but not others. Understanding who should be key stewards of a system might be considered through the lens of subsidiarity—which suggests that decisions and actions affecting interests should be carried out at lowest levels of organization possible, with the capacity to do so (McCay and Jentoft 1996; Marshall 2007).

Whether local actors—people and communities—have the motivations or capacity or not to take stewardship actions cannot be assumed, as it often is. As will be discussed below, stewardship is a phenomenon that depends on intrinsic and extrinsic motivations (e.g., ethics or incentives) and the capacity to act (e.g., assets and institutions), which can be differentiated by individuals and groups. Varying circumstances will influence both whether and how individuals, groups, or multi-stakeholder partnerships and networks mobilize to carry out stewardship actions. Thus, it can be helpful to understand the characteristics of (e.g., levels of resource dependence, socio-economic status, race, gender, etc.) and institutional, economic and social barriers facing different actors or groups and how these relate to stewardship motivations, capacity and actions (Henderson et al. 2014). Stewardship is also a fluid phenomenon that can change over time—as incentive structures, social norms, levels of dependence on resources, or access to resources and rights may change, individual actors or groups of actors may gain or lose the will and/or the ability to act as stewards.

### The Capacity to Steward: Local Assets and Governance

A fundamental concern of stewardship is capacity—i.e., whether individuals or communities are able to steward their resources. We suggest that there are two central factors that influence, positively or negatively, the capabilities of would-be stewards to take action: (1) local community assets and (2) broader governance factors.

First, the capacity of local communities to take stewardship actions is enabled or constrained by the presence or absence of local assets, which provide the resources or capabilities that can be mobilized to take action (Sen 1984; Allison and Ellis 2001). For example, research has suggested that factors such as infrastructure, technology, financing, levels of wealth or poverty, rights, knowledge, skills, leadership, and good relations can all support the capacity of communities to take stewardship action (Chapin et al. 2010; Gutiérrez et al. 2011; McConney et al. 2014). Yet, a more systematic consideration of assets could help to more clearly indicate how different assets influence stewardship. To this end, we draw on the literature on the set of capital assets listed in the sustainable livelihoods and community development literatures to propose a categorization that includes six assets that might be used to analyze local stewardship capacity: social capital (i.e., relationships, trust, networks), cultural capital (e.g., connections to place, traditions, knowledge, and practices), financial capital (e.g., income, credit, debt), physical capital (i.e., infrastructure and technology), human capital (e.g., education, skills, and demographics) and institutional capital (e.g., empowerment, agency, and options) (Scoones 1998; Allison and Ellis 2001; Green and Haines 2008; Bennett 2010; Bennett et al. 2012) (see Table 1 for definitions).

**Table 1:** Categories of assets that provide capacity to enable local environmental stewardship

Stewardship assets	Description
Social capital	The informal and formal relationships, including friendship, kinship and occupational networks, which facilitate trust and reciprocity to support stewardship
Cultural capital	The presence of and processes to maintain connections to place, traditions, knowledge, practices and artefacts that are central to a group’s identity and that support stewardship
Financial capital	The financial resources (e.g., income, credit, debt, wealth, and poverty) that are available to individuals or collectives (groups or communities) and provide the ability and means to take stewardship actions
Physical capital	The technologies (both traditional and modern) and other infrastructure that enables individuals and groups to steward living and physical resources
Human capital	The individual and group attributes, such as education, knowledge, leadership, past experiences, awareness, skills, and demographic factors (e.g., age and health of population) that enable stewardship
Institutional capital	The empowerment, agency, and options available to local communities to steward resources that results from broader governance, including systems of institutions (i.e., laws and policies, formal and informal organizations and decision-making processes) and structural processes related to power and politics (i.e., economic inequality, discrimination, levels of exclusion)

Second, governance—including systems of institutions (i.e., laws and policies, formal and informal organizations, and decision-making processes (Lockwood et al. 2010)) and structural processes related to power and politics (i.e., economic inequality, discrimination, exclusion from decision-making)—can empower or constrain the sense of agency, available options and capacity of would-be stewards (McLaughlin and Dietz 2008; Robbins 2012). For example, focusing on the context of small-scale fisheries, local stewardship efforts can be supported by national laws or policy frameworks that protect local fisher’s rights and tenure, formalize local fishers’ stewardship responsibilities, or that provide resources to support local community efforts to steward their own resources (Soliman 2014; FAO 2015). On the other hand, even when local small-scale fishers want to take action locally, the broader policy landscape may undermine their efforts by creating bureaucratic challenges or failing to recognize active or historical local stewards (Ayers and Kittinger 2014; Bennett et al. 2014). The presence, structure, and procedural norms of organizations—including formal government agencies, NGOs, local organizations, co-management bodies, or informal networks—can provide reinforcement for local collective actions, generate resources or facilitate learning for stewardship (McConney et al. 2014; Trimble et al. 2014; Medeiros et al. 2014). When external programs are introduced that do not align with local efforts this can crowd out local initiatives (Murtinho et al. 2013; Jupiter 2017). Procedural considerations, such as inclusion of stakeholders, participation in planning, social learning, knowledge co-production, cooperative management, trust building, negotiation, and conflict resolution, can also enable the effective stewardship of resources (Lockwood et al. 2010; Jupiter et al. 2014; McConney et al. 2014; Turner et al. 2014). Moreover, this past research demonstrates that local actors and communities can be empowered to steward local resources or their agency can be undermined by governance processes (e.g., top-down, co-managed, or bottom-up governance) or by structural power differentials or inequalities. We refer to the resultant level of empowerment and agency within local communities as institutional capital.

Yet local assets and supportive governance alone are insufficient—as they might be applied in support of actions that facilitate or that undermine stewardship. For example, in fisheries, more advanced or innovative technology (physical capital) might function as a “double-edged sword” leading either to overfishing (e.g., through more efficient gears) or to more sustainable harvesting of resources (e.g., through gears that reduce by-catch) (Finkbeiner et al. 2017). Similarly, access to additional financial resources might be used to develop alternative livelihoods thus reducing pressure on resources or be re-invested in increased capacity and intensification of fishing activities (Allison and Ellis 2001; Torell et al. 2010). Moreover, the mere presence of capacity and agency does not guarantee that actors will steward resources. As discussed below, individuals and communities with sufficient capacity need also to be motivated to pursue stewardship actions.

### Motivations: The Rationale and Will for Stewardship

Even when adequate capacity is present, some individuals or groups choose to steward resources while some do not. What, then, drives people or groups to take stewardship actions? Stewardship motivations might be defined simply as the reasons or incentive structures that drive people to take action to care for the environment. The literature on motivations and stewardship is vast. For our purposes, it is useful to engage with two broad analytical categories—intrinsic and extrinsic motivations—under which the array of previously discussed motivations for stewardship might be subsumed (Ryan and Deci 2000a; Cetas and Yasué 2017) (Table 2).

**Table 2:** Categories of intrinsic and extrinsic motivations for engaging in environmental stewardship

Types of motivations	Definition	Sub-categories of motivations for environmental stewardship
Intrinsic motivations	Intrinsic motivations are associated with actions that are expected to bring personal pleasure or satisfaction	Alignment with underlying ethics, morals, values, and beliefs
		Psychological needs for self-determination or self-actualization
Extrinsic motivations	Extrinsic motivations are associated with the expected achievement of separable outcomes	Perceived balance of direct costs and benefits of stewarding natural resources
		External rewards or sanctions, including economic, social, physical or legal

Intrinsic motivations are associated with actions that are expected to bring personal pleasure or satisfaction, through the achievement of psychological needs such as self-acceptance, feelings of competence or self-efficacy, sense of autonomy or wellbeing, and the need for belonging or affiliation with a group (Ryan and Deci 2000a; Tabernero and Hernández 2011). In the context of local environmental stewardship, we suggest two subcategories of intrinsic motivations: (a) underlying ethics, morals, values and beliefs and (b) a need for self-determination or self-actualization. First, people can be intrinsically motivated by their ethics, morals, values and beliefs. As Worrell and Appleby (2000) succinctly put it “…the ethical aspects of stewardship…provide an explicit, rational, moral underpinning for our treatment of natural resources and the natural world”. The idea of stewardship based on an underlying ethic has been examined extensively in environmental philosophy (Welchman 1999; Fernandes and Guiomar 2016). Take, for example, the classic “A Sand County Almanac” wherein Aldo Leopold argues eloquently for a “land ethic” (Leopold 1966) and similar volumes focused on the marine environment such as “The Sea Around Us” (Carson 1951) and “Values at Sea” (Dallmeyer 2003). These and similar texts suggest that an ethic of care, which is rooted in connections to non-human species, environments or special places, will motivate people to take stewardship actions. A stewardship ethic might also be derived from a person’s sense of moral responsibility to a god or other higher power to care for creation (Dyke et al. 1996), a sense of responsibility for a piece of land or resource (Berkes 1999; Ryan et al. 2003), altruistic concerns for current or future generations (Bourdeau 2004; Robinson et al. 2012), or an understanding of what constitutes a right relationship with others or the natural world (Chan et al. 2016). Simply put, actors might take stewardship actions because it is intrinsically motivating to do what is perceived to be the right thing.

Second, stewardship actions can also be intrinsically motivated by the desire for autonomy, relatedness, and competence—which correspond with the three universal psychological needs of self-determination theory (Ryan and Deci 2000b; Cetas and Yasué 2017)—and the higher order need for self-actualization (Maslow 1943). Autonomy refers to the desire to be able to affect one’s own future, relatedness is about feeling connected or belonging to a group, and competence refers to the feeling of being able to act and to achieve one’s goals. The idea of self-actualization is that the ultimate human aim is to be able to learn and grow and become one’s most accomplished self. Themes related to these concepts can be found across the literature on stewardship. For example, autonomy comes up in two ways: (1) Stewards can often be motivated to ensure the sustainability of resources so to maintain cultural or livelihood autonomy (Bennett et al. 2010) and (2) Stewardship programs that undermine the autonomy of resource users or land-owners may be opposed (Sorice et al. 2013). Other research has shown that environmental volunteers are often motivated by wanting to belong to a social group (Measham and Barnett 2008; Asah and Blahna 2012) and local stewards can be motivated by their affiliation with a community or group, such as farmers, fishers, hunters, or Indigenous groups (Silva and Mosimane 2014). A study by Ryan et al. (2003) shows that farmer’s are motivated to demonstrate a level of competence in caring for a resource and Bramston et al (2011) show that participation in environmental stewardship networks in rural Australia is motivated by a sense of belonging, care for the environment, and personal learning. While autonomy, relatedness, competence, and self-actualization focus on the individual, at the community level, similar framings for these intrinsic motivations might include the desire for community agency, collective solidarity, empowerment, identity or pride in collective achievements.

Extrinsic motivations on the other hand are associated with the expected achievement of separable outcomes, such as social reinforcements or economic benefits that are external to the self. Here we categorize extrinsic motivations as (a) the perceived balance of direct costs and benefits of stewarding natural resources and (b) externally provided rewards or sanctions which can be economic, social, physical or legal. First, stewards can be extrinsically motivated by the perceived direct lost opportunity costs (e.g., time, money) and instrumental benefits of stewarding resources. For example, farmers might be wary of the lost economic benefits associated with increasing a buffer along a stream just as fishers are often opposed to the creation of marine protected areas that restrict their ability to fish. On the other hand, the potential instrumental benefits that motivate environmental stewardship include direct economic benefits stemming from increased productivity, increases in provisioning, regulating, and supporting ecosystem services or improved health and well-being (Ryan et al. 2003; Grafton et al. 2006; Lopes and Videira 2013).

Second, external rewards and sanctions that can motivate stewardship include economic, social or legal factors. Economic motivations, which have received significant attention (Wunder 2007; Sorice et al. 2013), include financial rewards (e.g., payments to enable certain management actions, payments for ecosystem services, market premiums for more environmentally sustainable products) or financial disincentives such as fines or loss of access to markets. The desire for social recognition or avoidance of sanctions, which are both related to group norms and collective orientation, are often strong motivators for conservation of resources or for following rules set by a group (Basurto et al. 2016). Social recognition can take the form of praise, awards or certification and maintenance of good relations with other resource users. Social sanctions include declines in social capital with other members of a group or in some places the loss of property or gear, physical violence by other resource users, or being socially isolated or ostracized from the group (Acheson 1975; Hauzer et al. 2013). Finally, legal mechanisms (including customary laws) can be significant motivators—either through clearly articulating the societal norms and expectations as duties and responsibilities or through the use of legal sanctions and enforcement mechanisms (Gandiwa et al. 2013; Soliman 2014).

In short, intrinsic and extrinsic motivations can provide will (i.e., energy and persistence) and influence the choices and direct the actions chosen by stewards. They help to define the “of what?”, “why?” and “for what or whom?” of stewardship and to delineate the duties, obligations, and responsibilities of the steward. In general, a complex combination of intrinsic and extrinsic motivations work in concert to promote stewardship actions (Stern et al. 1993; Tabernero and Hernández 2011; Asah et al. 2014; Krasny et al. 2014). Some types of motivations, however, might have a stronger influence than others. For example, Asah and Blahna (2012); Asah et al. (2014) show how personal and social motivations are stronger predictors of people’s participation in volunteer urban stewardship activities than environmental rationales. Furthermore, intrinsic motivations might be more durable than extrinsic ones for promoting environmental action (Ryan et al. 2003; Cecere et al. 2014; Cetas and Yasué 2017). Motivational crowding out can occur when extrinsic incentives (e.g., monetary payments for stewardship, payments for ecosystem services) are applied in contexts where strong intrinsic motivations for stewardship already exist (Rode et al. 2015; Sorice and Donlan 2015). Thus, it is important to understand the array and strength of different motivations that actors in different contexts might have to engage in environmental stewardship.

### Stewardship Actions: Protection, Care or Sustainable Use

Taking action is the central focus of any discussion of environmental stewardship. Stewardship actions are the suite of approaches, activities, behaviors, and technologies that are applied to protect, restore or sustainably use the environment. The stewardship actions of local actors can emerge informally during day-to-day decision-making, can stem from formal or informal decision-making processes involving local collectives or networks, or can result from formal top-down processes or mandated requirements of government. Likewise, stewardship actions can derive from direct objectives relating to environmental sustainability, or indirectly as an ancillary effect of other objectives (i.e., livelihood security or social justice). Stewardship actions can occur at different scales, can address issues that are of greater or lesser complexity, and are taken by different individuals or groups of actors because of their motivations and available capacities. Below, we briefly discuss examples of the types of stewardship actions that might occur at different scales and levels of complexity.

Different stewardship actions may be taken to address problems of greater or lesser ecological or social–ecological complexity. Stewardship actions can be targeted for individual species, multiple species, individual habitats, entire ecosystems, or even integrated human-environment systems at scales ranging from neighborhoods to landscapes. For example, these actions might include limiting the harvest of a single recreationally, commercially, and culturally important species (Groesbeck et al. 2014), the establishment of no take terrestrial parks or marine protected areas to protect a species or habitat (Micheli et al. 2012), the active restoration of degraded habitats through replanting stream buffers (Sheppard et al. 2017), the practice of traditional comprehensive watershed management from mountaintops to the near-shore marine environment to protect ecosystems (Kaneshiro et al. 2005), the creation and management of urban green spaces or community gardens (Krasny and Tidball 2012), or the strategic reduction of dependence on resource-based livelihoods to decrease harvests (McCay et al. 2014). Stewardship can also take the form of passive management—leaving an area to regenerate—or simply choosing to not harvest from an area. In other words, stewardship might be accomplished through purposeful inaction. We do not pre-suppose the types of actions that constitute stewardship—and encourage a view of stewardship that looks beyond western conceptualizations of conservation and is inclusive of indigenous world-views and approaches (Berkes 1999; Brosius and Russell 2003; Hunn et al. 2003).

Stewardship actions can occur at different scales from local to macro scales. As an example of stewardship at the local scale, individual landowners might restore habitat on their land or a community might conserve a local forest or a coral reef. At the meso-scale, stewardship might take the form of protected land-scapes or sea-scapes—for example, through the creation of biosphere reserves (Reed 2016) or marine conservation planning that includes social and ecological considerations (Ban et al. 2013). Broader scale stewardship actions might be taken at national, eco-regional scale, or even at transboundary or regional scales—for example, this is the case with the planning of the Yellowstone to Yukon protected area and wildlife planning initiative (McGregor 2003) or regional marine conservation efforts such as the Coral Triangle Initiative (Walton et al. 2014).

These different stewardship actions can have impacts across scales and, in particular, local stewardship can be undermined or supported by stewardship actions taken (or not taken) in other places or at higher scales. Pulling invasive species from a single farm may do little good if not supported by actions in the surrounding landscape. Similarly, in inherently complex systems, specific stewardship actions (or lack thereof) can have unintended “cross-scale” benefits or consequences for other actors, system components, or systems (Gunderson and Holling 2002; Bunce et al. 2010; Larrosa et al. 2016). For example, the local retention of benefits from sustainable use of a forest resource is more likely than from a marine protected area designed to protect a migratory fish species. The latter example may instead benefit others who are further away. On the contrary, a coral reef ecosystem might be impacted by upstream farming practices that fail to deal with erosion or agricultural run-off (Álvarez-Romero et al. 2011; Bégin et al. 2016). In sum, to comprehend the nature and effectiveness of local stewardship, it is critical to analyze the scales where stewardship actions are taking place, cross-scale interactions and whether stewardship action is occurring at the relevant scale to achieve the desired ecological and social outcomes.

While our focus here is on direct stewardship actions, some activities that are labeled environmental stewardship operate indirectly. These stewardship supporting activities might include activities such as environmental education of resource users or youth (Stern et al. 2008; Tidball and Krasny 2011), transmission of traditional ecological knowledge (Bussey et al. 2016; Reo et al. 2017), network building activities (Alexander et al. 2015; Blythe et al. 2017), environmental governance or policy reforms (Gelcich et al. 2010), systems of rewards and punishments (Ostrom 1990; Hauzer et al. 2013), and scientific or participatory monitoring and research (Shirk et al. 2012; Silva and Krasny 2016). Activities such as these are fundamental to local stewardship; however, these activities alone do not improve the environment. The premise is that through promoting motivations and augmenting capacity these activities can indirectly encourage and enable the direct actions of actors to protect, restore or sustainably use the environment. Stewardship supporting activities can be implemented by local groups, or as discussed later, instigated by external organizations.

### The Social–Ecological Context of Stewardship

We define social–ecological context as the broader set of social, cultural, economic, political and biophysical factors occurring beyond the local system of study. The broader social–ecological context influences local stewardship efforts in two ways. First, stewardship capacity is influenced by the speed, scale, severity, complexity, and predictability of the social and ecological changes that are occurring and how these impact social and ecological aspects of local systems. This framing builds on both resilience (Holling 2001; Lebel et al. 2006; Walker and Salt 2006; Berkes and Ross 2016) and governability (Chuenpagdee and Jentoft 2009; Kooiman and Bavinck 2013) literatures, which suggest that adaptive and governance capacity needs to be understood within the broader socio-economic, environmental, and governance context. For example, the impacts of climatic change can severely impact resources and people’s ability to respond proactively (Kalikoski et al. 2010; Marshall 2016). Communities are constantly confronted with a number of other social, economic, political, governance, and biophysical drivers of change occurring at higher scales that might challenge stewardship efforts (Tuler et al. 2008; Bennett et al. 2015a; Moshy et al. 2015). Barratt and Allison (2014) highlight how vulnerability to environmental change can undermine community management of natural resources through a case study of Lake Victoria. Yet, not all changes are negative and change can also support community stewardship efforts—for example, the resurgence of external market interest in Community Supported Fisheries or Community Supported Agriculture can incentivize local management (Brinson et al. 2011).

Second, the broader social–ecological context determines which stewardship actions will be socially, culturally or politically feasible, appropriate or effective. In different cultural contexts the types of stewardship actions that will be deemed appropriate will differ (Gavin et al. 2015; Ens et al. 2016). For many indigenous communities whose cultural identity and harvesting practices are deeply interconnected, the idea of “no-take” conservation may be antithetical to their holistic “social-ecological” worldview (Berkes 1999). Additionally, in a context where local cultural identity depends on the harvest of certain mega-fauna (e.g., sea turtles, whales, caribou, polar bear), the complete closure of these areas to harvesting (even when species are considered vulnerable or endangered) may be deemed unacceptable (Clark et al. 2008). Considering what might constitute due and appropriate process for promoting management or conservation interventions in different socio-political or governance contexts is also important. Externally driven stewardship actions may be considered a form of “green grabbing” or “ocean grabbing” when the process of implementation undermines local autonomy or sovereignty in the process (Corson and MacDonald 2012; Bennett et al. 2015b). Negative perceptions of governance and decision-making can lead to opposition to conservation or management and, in effect, discourage stewardship (Gelcich and O’Keeffe 2016). Thus, it can be instructive to understand the extent to which stewardship actions and decision-making process align with or fit the local social and ecological context (Wilson 2006; Epstein et al. 2015).

### The Outcomes of Stewardship

Stewardship is for naught if it is not producing desirable ecological and social outcomes. Environmental objectives may be a primary motivator for engaging in stewardship—for example, improving the sustainability of resources, restoring degraded habitats, recovering wildlife, increasing fish stocks or preserving a wilderness area. However, these environmental objectives are often directly linked to or associated with desired social outcomes, which might be social, cultural, economic, health, physical or governance-related (Donatuto et al. 2014; Biedenweg et al. 2016; Breslow et al. 2016; Kaplan-Hallam and Bennett 2017). Social objectives also include process considerations—e.g., how stewardship decisions are made and the roles that different actors play in stewarding the resource (Jupiter et al. 2014; Bennett and Dearden 2014). Local resource users and communities may pursue both ecological and social objectives simultaneously (Kittinger et al. 2016).

Thus, analysis of the outcomes of environmental stewardship should seek to understand how stewardship affects both ecological and social aspects and whether the outcomes of stewardship match with desired objectives. Given that stewardship occurs in complex social–ecological systems, attention is needed to feedbacks, synergies and trade-offs between social and ecological considerations in stewardship planning processes and in monitoring and evaluation frameworks (Chan et al. 2006; Kareiva et al. 2007; Oteros-Rozas et al. 2013). Additional considerations when seeking to understand the full impact of environmental stewardship requires inquiry into: (a) both the intended and unintended consequences of stewardship actions (Larrosa et al. 2016), (b) the potential benefits that occur beyond the environmental stewardship schemes remit (Courtney et al. 2013), (c) the distribution of the costs and benefits of stewardship initiatives between different groups (Pascual et al. 2014), and (d) the impacts of initiatives across spatial and temporal scales and for both current and future generations (Chan and Satterfield 2013). Understanding the extent to which outcomes match objectives and produce other (positive or negative) outcomes provides feedback for evaluating and adapting local stewardship approaches or to aggregate lessons learned and improve broader policies and programs intended to improve stewardship. Additionally, demonstrably positive outcomes from stewardship may be necessary to establish the legitimacy of local stewardship efforts.

### A Definition and Analytical Framework for Environmental Stewardship

**Fig. 2:**
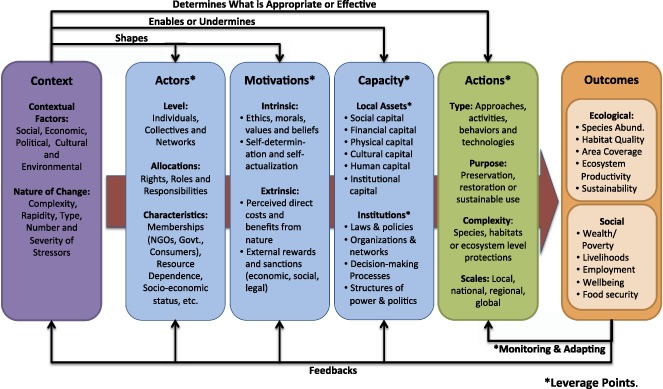
Analytical framework for the elements of local environmental stewardship. Strategic interventions – government policies, NGO programs or market mechanisms—can be applied at different leverage points (*) to support or promote local environmental stewardship efforts

In sum, we bring these various elements together in an integrative conceptual framework for environmental stewardship (Fig. 2)—in order to provide a structure for analysis, a common language to stimulate further engagement, and a guide for efforts aimed at strategically promoting environmental stewardship. The different elements of the framework come together as follows: Stewardship actions are the suite of approaches, activities, behaviors, and technologies that are applied to protect, restore or sustainably use the environment; Individuals, groups or networks of actors initiate and take stewardship actions; Intrinsic and extrinsic motivations determine the rationales, moral obligations, and willpower for taking stewardship actions; Capacity, which is determined by both local assets and broader governance, influences the ability of local actors to engage in stewardship actions; Broader social and ecological contextual factors, including the speed and complexity of change, can support or undermine stewardship capacity and determine what actions will be appropriate and/or effective; and these factors converge to enable or undermine actions and to produce social and ecological outcomes. We provide separate definitions for each of the elements of environmental stewardship in Table 3.

**Table 3:** Definitions of key concepts related to environmental stewardship

Elements of stewardship	Definitions
Stewardship actions	The approaches, activities, behaviors, and technologies applied to protect, restore or sustainably use the environment. Stewardship actions can occur at different scales, can address issues that are more or less complex, and are taken by different actors or groups based on their characteristics, motivations, and capacities
Actors (or stewards)	The different individuals or configurations of stewards across scales of organization who are driving stewardship initiatives. Actors have different actual and desired rights, roles, and responsibilities. Actor characteristics may influence willingness, motivations, and ability to participate in stewardship
Motivations for stewardship	The intrinsic or extrinsic incentive structures or reasons that people take action to care for the environment. Intrinsic motivations are associated with actions that are expected to bring personal pleasure or satisfaction, through (a) alignment with ethics, morals, values, and beliefs or (b) the achievement of psychological needs for self-determination and self-actualization. Extrinsic motivations are associated with the expected achievement of separable outcomes including (a) the perceived direct costs and benefits of stewarding resources and (b) externally provided rewards or sanctions. An individual or group’s motivations defines the rationale for actions, clarifies obligations and provides the willpower to act
Capacity for stewardship	The ability to take action to care for the environment. The capacity of actors to take stewardship actions is enabled or constrained by local assets and broader governance factors. Local assets that support stewardship capacity can include social, financial, physical, cultural, political human, and institutional capital. Broader governance—including institutions (i.e., laws and policies, organizations and networks, and decision-making processes) and structural processes related to power and politics (i.e., economic inequality, discrimination, exclusion from decision-making)—might also empower or constrain the agency, options and capacity of stewards
Context of stewardship	The set of social, cultural, economic, political, and biophysical factors that determines which stewardship actions will be socially, culturally or politically appropriate and ecologically effective. The nature of change, including complexity, scale, speed, type, and severity, occurring can challenge local stewardship capacity
Outcomes of stewardship	The ecological and social impacts of stewardship actions. The outcomes of stewardship can be intended or unintended, produce synergies or trade-offs, be desirable or undesirable, and have differential costs and benefits for distinct groups
Stewardship interventions	The policies, programs or market mechanisms that different organizations and actors—including governments, NGOs, interest groups, and local communities—promote and implement with the intention of enabling or developing environmental stewardship
Leverage points for stewardship	The specific levers or points where different local or external organizations and actors might intervene to produce change in the stewardship of a system in order to facilitate desirable ecological and social outcomes. Leverage points can include introducing new actors, providing incentives, augmenting capacity or governance, promoting certain actions, or monitoring outcomes to facilitate adaptive management

## Supporting and Researching Local Environmental Stewardship

Having set out a framework, we now briefly examine how different organizations might use it to guide interventions aiming to support or promote local stewardship and also how it might be applied in future research efforts.

### Interventions and Leverage Points for Stewardship

Different organizations—including governments, NGOs, and the private sector—and individuals often attempt to develop or support pre-existing environmental stewardship efforts by local people. To do so, these external groups promote and implement specific policies, programs and market mechanisms—which we call “interventions” here—to support or enable local stewardship potential and improve outcomes through different “leverage points”. Leverage points is a term which refers to the levers or places in a system where a strategic shift can produce changes in the entire system (Meadows 2009). Our preliminary analysis suggests five primary leverage points in the framework (see * in Fig. 2) where many governmental and non-governmental organizations attempt to promote environmental stewardship through interventions that: (1) introduce new actors, (2) provide incentives, (3) augment local capacity or institutions, (4) promote or support the implementation of specific actions, or (5) monitor and evaluate the outcomes of stewardship to facilitate adaptive management.

For example, many education programs and social marketing campaigns may seek to change people’s mental models or alter *intrinsic motivations* through creating connections with nature and changing people’s ethics, values or beliefs (McKenzie-Mohr et al. 2011; Leisher et al. 2012). Payments for environmental service (PES) programs were originally designed to provide external financial rewards for engaging in stewardship (Wunder 2007), thus targeting *extrinsic motivations*, though PES programs are becoming more nuanced in how they are designed to match a variety of local motivations (Rode et al. 2016). Some stewardship programs focus on building stewardship networks, at times introducing new *actors* or organizations to facilitate these processes (Kowalski and Jenkins 2015; Jenkins et al. 2017). Sustainable livelihoods programs aim to build local capacity for environmental stewardship (Cattermoul et al. 2008; Bennett 2010). Programs that advocate for recognition of local rights (i.e., rights-based approaches) or property rights or the creation of higher-level policies that recognize and support local stewardship are intervening at the level of institutions (Georgakopoulos et al. 2008; Gilmour et al. 2012). Some conservation organizations often simply promote specific *actions*—for example, the creation of more marine or terrestrial protected areas, the use of stream buffers in farming to protect streams, etc. Many real-world interventions focus on more than one leverage point simultaneously—for example, the Fish Forever program that is promoted by Rare and Environmental Defense Fund combines environmental education and outreach, property rights, capacity supports for technical management with specific actions (Fish Forever 2017)—and many programs are getting more holistic and comprehensive over time. Yet, the leverage point(s) being targeted through different interventions, and how these interact with other elements of stewardship, are often not explicitly articulated by government policies or NGO programs (Foale et al. 2013). This is surprising as many of the interventions focus not on promoting specific actions but rather on stewardship supporting activities.

The overall effectiveness and appropriateness of the myriad interventions and associated leverage points is a matter of ongoing debate, which requires more space than we can devote to it here. Suffice it to say that all stewardship interventions should be considered a “work in progress”, which require continual monitoring, evaluation and adaptation. The effectiveness of these different interventions and leverage points needs to be better understood and tested empirically, to understand whether they are actually supporting or undermining local stewardship efforts. The above discussion also highlights the importance of understanding the local context and clearly articulating and continually revising a “theory of change” for all externally promoted interventions that seek to promote stewardship.

### Future Applications of the Stewardship Framework

The analytical framework that we provide here might be applied to future research that seeks to: (a) descriptively assess the elements of stewardship in case studies in different contexts, (b) guide decision-making and the design of environmental stewardship initiatives or interventions, (c) evaluate the effectiveness of local initiatives or external interventions that seek to promote stewardship, and (d) delve more deeply into questions related to specific aspects of stewardship to provide crucial theoretical and practical insights. We discuss each of these briefly below.

#### Descriptive assessments of stewardship in different contexts

The descriptive analysis of localized environmental stewardship efforts in different contexts can help researchers, local stewardship groups and/or external organizations to understand the configuration of the different elements of stewardship. For example, one might find that local communities are highly effective at conserving local resources and thus that their efforts should be recognized and supported by external organizations rather than undermined through the imposition of external models of conservation (Jupiter 2017). Conversely, local community groups may have strong motivations to take stewardship actions but may simply lack the capacity to do so (Bennett et al. 2014; Barratt et al. 2015). However, accurate analysis of stewardship in different contexts may require extended engagement to get a complete picture of how the different elements of stewardship come together. In the case of traditional resource harvesters, different motivations for stewardship are co-constituted with culture, customs, harvesting practices, and traditional knowledge, manifested in group norms and rules of engagement and emerge as linked use and management actions (Berkes 1999; Reo and Whyte 2011). Analysis of case studies can help to build a corpus of research on the topic, might inform local deliberations in other locations on how to (re)design local stewardship actions or could help to guide the investments of external organizations who are interested in investing in environmental stewardship in different locales.

#### Prescriptive analysis to aid design and decision-making

By strengthening environmental stewardship, it is hoped that communities will be able to foster a virtuous circle of improved environmental management and social welfare. One of our aims in proposing this framework is to aid in the integration of stewardship considerations into planned or anticipated interventions, and to provide the basis for making recommendations for the types of interventions likely to be most beneficial (i.e., should we increase capacity, improve institutions or leverage motivations?) in different contexts. For example, when interventions are made by outside organizations, care must be taken not to undermine pre-existing institutions or cooperation between actors by targeting specific levers as if they were merely a resource or a means for external organizations to meet their own goals and motivations of environmental conservation. This does not mean that attempts to intervene, support, leverage and, where necessary, promote local stewardship should be abandoned. However, we urge cautious and mindful engagements as there are no panaceas.

In particular, it can be critical to understand the local context, including the level to which stewardship already exists and the current configuration of the different elements (actors, capacity, motivations) of stewardship, to ensure that outside efforts are aligned with local efforts, realities, and aspirations. Recent attention to motivations, and related concepts, has stressed the need for alignment of conservation policy incentives with local ethics, values, norms, and motivations (Chan et al. 2016; Nyborg et al. 2016; Lubchenco et al. 2016). Murtinho et al. (2013) show that external funding is often necessary for stewardship but is only beneficial when it is asked for rather than offered. Careful consideration is also needed to minimize any negative impacts of stewardship actions on the most vulnerable and marginalized groups within these communities (Mansuri and Rao 2004), and to ensure that the responsibility to steward is not expected from individuals or groups who do not have the capacity to carry out such actions, or who might experience costs that are greater than benefits. The genuine inclusion of local communities in decision-making and stewardship practices has the potential, if done well, to help improve the fit of stewardship interventions and increase the likelihood of success. We highlight the potential of participatory methods of engagement, human-centered design thinking, and adaptive co-management for innovating in the design of stewardship programs (Evans et al. 2006; Reed 2008; Armitage et al. 2010; Chevalier and Buckles 2013; Sorice and Donlan 2015; Gelcich and Donlan 2015; Romero Manrique de Lara and Corral 2017).

#### Evaluating the effectiveness of local stewardship initiatives, external interventions, and associated leverage points

The effectiveness of local stewardship can be improved through monitoring and evaluation, either by scientists or through participatory processes (Driscoll et al. 2012; Silbernagel et al. 2015; Silva and Krasny 2016), and subsequently adapted based on this knowledge (Armitage et al. 2010; Plummer et al. 2012). As discussed above, in all environmental policy realms, there is an array of external interventions that target different leverage points to promote and facilitate environmental stewardship. Yet it is often unclear the extent to which these different programs, policies or market mechanisms are effective at enhancing stewardship outcomes. There is thus a need to monitor and evaluate the effectiveness of both local initiatives and external interventions as well as to understand the impacts of focusing efforts on different leverage points (motivations, capacity, governance, etc.) in different contexts. This research can build on past research that focuses on specific elements—such as actors, actions, local capacity, governance or motivations—and synthesize these findings to better understand the effects of different elements on stewardship outcomes. The insights from evaluations can be applied to adaptively manage stewardship interventions, revisit an organization’s “theory of change,” and even to re-formulate entire interventions when found to be ineffective or guide strategic investments of external organizations.

#### Further research to develop theoretical or practical insights

Finally, the framework that we have provided here might serve as a guide for more systematic analysis to develop practical insights or targeted theoretical inquiries into the individual elements and their relation to overall environmental stewardship. Practically, there is a need to better understand what factors or combinations of factors are enabling or inhibiting the success of environmental stewardship. The framework that we provide can aid in the systematic analysis of how contextual factors, intrinsic and extrinsic motivations, and the various elements of local capacity or institutions influence the stewardship choices of actors and their respective effectiveness. The application of this framework across a suite of research case study sites would enable comparison across sites and the scaling up of insights to develop more generalizable insights or lessons learned to guide future initiatives. Theoretically, there is a need for continued research on and testing of hypotheses around many of the elements of the stewardship framework.

## Conclusion

The global scale of many current environmental issues might lead to the perception that targeting local environmental stewardship could no longer meet these challenges. However, environmental stewardship is one way through which people get involved in promoting sustainability. This paper addresses a gap in the literature by articulating a definition and presenting an integrative analytical framework that encompasses important elements of local environmental stewardship. The framework is applicable to different social and ecological contexts. A common language for the elements of stewardship is proposed to stimulate further engagement while helping to build a more robust body of academic research and theory on environmental stewardship. This more comprehensive understanding and analytical framework for environmental stewardship will also provide important practical insights into how to design and promote more meaningful and effective environmental policies and programs. Ultimately, our aim is to raise the profile of environmental stewardship as a valuable and holistic concept for guiding productive and sustained relationships with the environment.
